# Prognostic factors in the management of paediatric patients with retropharyngeal and parapharyngeal infections

**DOI:** 10.1017/S002221512610471X

**Published:** 2026-05

**Authors:** Kerry Hu, Maryam Sattar Othman, Jacob Davidson, Julie Strychowsky, M. Elise Graham, Murad Husein, Josee Paradis, Keng Yeow Tay, Andrew Leung, Peng You

**Affiliations:** 1 Schulich School of Medicine and Dentistry, University of Western Ontario, London, Canada; 2 Division of Pediatric Surgery, Schulich School of Medicine & Dentistry, Western University, London, ON, Canada; 3 Department of Otolaryngology—Head and Neck Surgery, Schulich School of Medicine & Dentistry, Western University, London, ON, Canada; 4 Division of Otolaryngology—Head and Neck Surgery, Dalhousie University, Halifax, NS, Canada; 5 Department of Medical Imaging, Victoria Hospital, London Health Sciences Centre, ON, Canada

**Keywords:** deep neck space infections, parapharyngeal abscess, paediatric infections, retropharyngeal abscess, surgical intervention

## Abstract

**Background:**

Deep neck space infections are uncommon but potentially life-threatening conditions with variable presentation in children without clear indications for medical versus surgical management. This study aimed to identify predictors of surgical intervention.

**Methods:**

A retrospective review was conducted of paediatric patients with deep neck space infections at a tertiary centre between 2014 and 2024. Patients were grouped by treatment approach: medical therapy, primary surgery or surgery following medical therapy. Clinical, radiological and outcome variables were analysed to identify predictors of surgical intervention.

**Results:**

Ninety-two patients were included: 51.1 per cent were managed successfully with antibiotics alone, and 48.9 per cent required surgery initially or after medical trial. Independent predictors of surgical intervention included younger age, fever (< 38.0° Celsius) at presentation, computed tomography confirmed abscess and cross-sectional area greater than 3.00 cm^2^.

**Conclusion:**

Most deep neck infections respond to medical management, though younger age, fever and larger abscess size should prompt consideration for surgical consideration.

## Introduction

Retropharyngeal and parapharyngeal infections are uncommon but clinically important deep neck space infections (DNSI), most frequently affecting younger children.^1^^,^^2^ They typically arise as complications of upper aerodigestive tract infections,^1^ presenting with nonspecific symptoms such as fever, neck stiffness, pharyngitis or odynophagia.^2^ These infections can progress rapidly, leading to airway compromise, mediastinitis, sepsis and vascular complications.^3^^,^^4^

Although surgical drainage has traditionally been considered the gold standard treatment,^4^^–^^6^ growing evidence has shown that medical therapy can be both safe and effective in clinically stable patients.^7^^–^^13^ Careful selection of candidates for antibiotics and supportive care allows patients to avoid surgical risk and its associated morbidity,^2^^,^^7^^–^^12^ while lowering overall healthcare utilisation and costs.^5^ Although computed tomography (CT) with contrast has become the cornerstone in diagnosis, its predictive value is not perfect.^9^^,^^11^^,^^14^ Ultimately, the lack of reliable prognostic markers has resulted in variability between institutions and among clinicians, leading to inconsistent care, potentially unnecessary procedures and delays in definitive treatment.^15^

Several studies have concluded that no symptom, sign or radiological feature can consistently predict treatment outcomes or surgical necessity.^3^^,^^12^ In response to this clinical ambiguity, the present study sought to investigate prognostic factors that are associated with treatment success or failure, with the goal of informing more objective, evidence-based decision-making in the management of paediatric retropharyngeal and parapharyngeal abscess.

## Materials and methods

A retrospective chart review was performed on paediatric patients (<18 years) presenting to London Health Science Centre (LHSC), a tertiary academic hospital in London, Ontario, Canada, between 1 January 2014 to 1 January 2024. Patients were included in the study if they were diagnosed with a retropharyngeal or parapharyngeal abscess, phlegmon or cellulitis and less than 18 years of age at the time of diagnosis.

Cases were identified using International Classification of Diseases 10^th^ Edition (ICD-10) codes J39.0 (retropharyngeal/parapharyngeal abscess) and J36 (peritonsillar abscess). Patients with J36 were screened and included only if they had a concurrent diagnosis of retropharyngeal or parapharyngeal abscess, phlegmon or cellulitis. Patients were excluded if they: (1) presented outside the study period, (2) had unrelated diagnoses, (3) received intra-venous (IV) antibiotic or surgical treatment at a different institution, and/or (4) had incomplete medical records. Institutional Review Board approval was obtained from the Health Sciences Research Ethics Board at Western University.

Data were extracted from electronic medical records and collected using REDCap, a secure data management platform,^16^^,^^17^ hosted at the London Health Sciences Centre Research Institute. Collected variables included patient demographics, clinical presentation, medical history, laboratory results at the time of presentation, imaging findings, treatment details, clinical course and outcomes. The primary outcome measure was the requirement for surgical drainage of DNSI. Secondary outcomes included length of hospital stay, complications (e.g., airway distress, mediastinitis) and recurrence. Diagnostic confirmation of DNSI was based on contrast-enhanced CT of the head and neck. Imaging findings included the location of the infection, the diagnostic category (abscess, phlegmon or cellulitis), the size of the collection and, if applicable, the presence of rim enhancement (RE). CT-identified infection size was recorded using the largest long axis measurement, and the cross-sectional area (CSA) was calculated from the largest two dimensions. For example, for an abscess measuring 3.00 cm by 2.31 cm by 0.52 cm, the largest long axis measurement will be 3.00 cm, and the CSA will be measured to be 3.00 cm × 2.31 cm.

Patients were grouped into those who achieved successful resolution of infection through medical management alone versus those who required surgery. Within the surgical group, patients were considered converted cases if they had initially received medical therapy but required surgical drainage later in their hospital course. Where relevant, analyses comparing primary surgical and converted patients were conducted to explore differences in baseline characteristics, predictors of treatment failure and post-operative outcomes of those who failed an initial trial of medical management.

Descriptive statistics were used to compare patient characteristics, clinical findings, management and treatment outcomes. Between-group comparisons for patients managed medically and surgically were calculated using chi-squared tests for categorical variables and Kruskal–Wallis test for continuous variables. An alpha value of 0.05 was used to determine statistical significance.

Multivariate logistic regression was used to determine the most significant prognostic factors for the need for surgical management. Significant variables in the bivariate analyses were used as baseline predictors in a multivariate logistic regression model. Initially, a stepwise model process was used to determine variables with the greatest predictive power. The final multivariable model was based on model parsimony and adequate goodness-of-fit statistics.^18^

## Results and analysis

### Patient demographics and medical history

A total of 92 patients met the inclusion criteria and were included in the study (Table 1). The median patient age was 4.5 years, with a range of 0.6 to 17.1 years. A larger proportion of patients were male (61.0 per cent). Within the study cohort, 48.9 per cent (n= 45) of patients received surgery, including a subset of 26 individuals who received an initial trial of medical therapy prior to surgery. Patients in the surgical group (SG) had a median age of 3.3 years and were significantly younger than those in the medical group (MG) at 5.1 years (*p* = 0.0018). There was no previous history of DNSI within the patient cohort. A recent upper respiratory tract infection, although not a significant finding, was more prevalent in patients treated surgically (50.0 per cent in SG vs. 29.6 per cent in MG). Most patients were antibiotic naïve on presentation, with similar distributions across both treatment groups. Immunisation status, as well as incidence of co-morbidities, were similarly distributed across both groups.

**Table 1.** Patient characteristics and past medical history Table 1 long description.

Parameter	Total	Medical group	Surgical group	*p*-Value
No. of patients, % (n)	100.0 (92)	51.1 (47)	48.9 (45)	
Median age (IQR)	4.5 (2.7−6.6)	5.1 (3.7−7.9)	3.3 (2.0−5.7)*	*p = 0.0018
Sex, % (n)	61.5 (56)	56.5 (26)	66.6 (30)	*p* = 0.3200
Male				
Female	38.5 (35)	43.5 (20)	33.3 (15)	
History of DNSI, % (n)				n/a
No	100.0 (90)	100.0 (46)	100.0 (44)	
Yes	0.0 (0)	0.0 (0)	0.0 (0)	
Recent URTI, % (n)				*p* = 0.0500
No	60.2 (53)	70.5 (31)	50.0 (22)	
Yes	39.8 (35)	29.6 (13)	50.0 (22)	
Prior antibiotic use, % (n)				*p* = 0.7140
No	73.8 (62)	72.1 (31)	75.6 (31)	
Yes	26.2 (22)	27.9 (12)	24.4 (10)	
Immunisation status, % (n)				*p* = 1.0000
Not immunised	6.4 (5)	5.1 (2)	7.7 (3)	
Partially immunised	1.3 (1)	2.6 (1)	0.0 (0)	
Immunised	92.3 (72)	92.3 (36)	92.3 (36)	
Underlying health conditions, % (n)				*p* = 0.1196
No	82.2 (74)	76.1 (35)	88.6 (39)	
Yes	17.8 (16)	23.9 (11)	11.4 (5)	

DNSI = deep neck space infection; IQR = interquartile range; URTI = upper respiratory tract infection.

* *p* < 0.05 (statistically significant).

*Note:* Immunisation status corresponds to age-appropriate vaccines based on the Ontario Immunisation Schedule^19^ and was assessed based on documented responses in clinical documents.

### Clinical presentation and laboratory findings

On presentation, clinical symptoms and physical examination findings were broadly similar between the MG and SG (Table 2). Fever, defined within this study as oral temperature measuring greater than 38.0° Celsius, was significantly more common for those who ultimately required surgery, whereas neck pain was negatively correlated with surgical treatment within our cohort. Across both groups, the most common physical finding was restricted neck mobility, followed by cervical lymphadenopathy. However, no physical examination features demonstrated statistically significant differences between treatment groups. Furthermore, there was no significant difference between the groups for duration of symptoms prior to initial presentation or laboratory findings.

**Table 2.** Symptoms on history and physical exam findings Table 2 long description.

Parameter	Medical group (n = 47) % (n)	Surgical group (n = 45) % (n)	*p*-Value
Described symptoms on history			
Fever*	68.1 (32)	91.1 (41)	^*^p = 0.0092
Restriction of neck range of movement	59.6 (28)	68.9 (31)	*p* = 0.3518
Decreased oral intake	46.8 (22)	53.3 (24)	*p* = 0.5315
Sore throat	34.0 (16)	42.2 (19)	*p* = 0.4192
Neck pain*	63.8 (30)	37.8 (17)	^*^p = 0.0125
Drooling	19.2 (9)	20.0 (9)	*p* = 0.9181
Odynophagia	34.0 (16)	22.2 (10)	*p* = 0.2081
Dysphonia	12.8 (6)	15.6 (7)	*p* = 0.7010
Dysphagia	8.5 (4)	11.1 (5)	*p* = 0.7369
Neck swelling	25.5 (12)	28.9 (13)	*p* = 0.7175
Respiratory distress	0.0 (0)	2.2 (1)	*p* = 0.4891
Other	40.4 (19)	55.6 (25)	*p* = 0.1464
Physical exam findings			
Cervical lymphadenopathy	57.5 (27)	55.6 (25)	*p* = 0.8549
Trismus	17.0 (8)	8.9 (4)	*p* = 0.2469
Pharyngeal bulging	31.9 (15)	44.4 (20)	*p* = 0.2159
Neck mass	21.3 (10)	17.8 (8)	*p* = 0.6724
Restricted neck mobility	66.0 (31)	64.4 (29)	*p* = 0.8789
Stridor	0.0 (0)	2.2 (1)	*p* = 0.4891
Other	27.7 (13)	42.2 (19)	*p* = 0.1426

* *p* < 0.05 (statistically significant).

### Imaging studies

Two patients were diagnosed clinically without imaging; one based on symptoms of respiratory distress in the SG, and another patient was treated medically based on neck stiffness and improved clinically; therefore, imaging was deemed unnecessary. These two patients were not included in the following analysis. The remaining patients underwent CT with contrast that showed an infection primarily in the retropharyngeal space (84.8 per cent) versus parapharyngeal space. Fifteen patients (16.7 per cent) also had a concurrent diagnosis of peritonsillar infection. Diagnoses were based on CT imaging reports, showing that abscess was the most identified at 53.3 per cent, followed by oedema (21.1 per cent), suppurative lymph node (13.3 per cent), phlegmon (7.8 per cent) and cellulitis (4.4 per cent) as shown in Table 3. The diagnosis category by CT imaging was significantly different (*p* = 0.0048) between the treatment groups.

**Table 3.** Imaging features: CT imaging Table 3 long description.

Parameter	Overall	Medical	Surgical	*p*-Value
	(*n* = 90)% (n)	(*n* = 46) % (n)	(*n* = 44) % (n)	
Diagnosis category				^*^p = 0.0048
Abscess	52.2 (48)	36.2 (17)	68.9 (31)	
Oedema	20.6 (19)	31.9 (15)	8.9 (4)	
Suppurative lymph node	13.0 (12)	10.6 (5)	15.6 (7)	
Phlegmon	7.6 (7)	10.6 (5)	4.4 (2)	
Cellulitis	4.3 (4)	8.5 (4)	0.0 (0)	

* *p* < 0.05 (statistically significant).

CT = computed tomography.

The size of the collection was assessed using CT-reported largest reported measurement and CSA as calculated by multiplying the largest two long-axis dimensions (Table 4). Overall, the largest long dimensional measurements ranged between 0.29 cm and 7.50 cm. Calculated CSA ranged between 1.63 cm^2^ and 17.25 cm^2^. Significant differentiation between MG and SG was found for infections greater than 2.50 cm^2^ (*p*= 0.0225). Within this study, RE was a commonly described finding at 83.3 per cent of all patients but not independently predictive of surgery after accounting for size.

**Table 4.** CT imaging characteristics of size, CSA and presence of rim enhancement Table 4 long description.

	Parameter	Overall (n = 90)	Medical (n = 46)	Surgical (n = 44)	*p*-Value
Largest long-axis dimension^†^					
	Median (IQR)	2.4 (1.8−3.0)	2.2 (1.7−2.8)	2.5 (1.9−3.4)	p = 0.0710
	> 2.00 cm, % (n)	67.3 (37)	59.1 (13)	72.7 (24)	*p* = 0.2910
	> 2.50 cm, % (n)	40.0 (22)	27.3 (6)	48.5 (16)	*p* = 0.1157
	> 3.00 cm, % (n)	23.6 (13)	9.1 (2)	33.3 (11)	*p* = 0.0533
	> 4.00 cm, % (n)	9.1 (5)	4.6 (1)	12.1 (4)	*p* = 0.6377
Cross-sectional area^‡^					
	Median (IQR)	3.6 (2.5−6.8)	2.6 (2.0−4.2)	5.0 (3.2−7.0)	*p* = 0.0134
	> 2.00 cm^2^, % (n)	83.6 (46)	72.7 (16)	90.9 (30)	*p* = 0.1340
	> 2.50 cm^2^, % (n)	76.4 (42)	59.1 (13)	87.9 (29)	^*^ *p =* 0.0225
	> 3.00 cm^2^, % (n)	61.8 (34)	40.9 (9)	75.8 (25)	^*^ *p* = 0.0092
	> 4.00 cm^2^, % (n)	47.3 (26)	27.3 (6)	60.6 (20)	^*^ *p* = 0.0153
Presence of rim enhancement					*p* = 0.1363
	No, % (n)	16.7 (9)	27.3 (6)	9.4 (3)	
	Yes, % (n)	83.3 (45)	72.7 (16)	90.6 (29)	

* *p* < 0.05 (statistically significant).

† Identified as largest reported measurement in computed tomography report.

‡ Calculated using largest two reported measurements from computed tomography report.

CSA = cross-sectional area; CT = computed tomography; IQR = interquartile range.

### Treatment details and clinical course

All patients received IV antibiotics throughout the course of their stay, with ceftriaxone being the most common agent in their treatment regimen (84.7 per cent). Nearly half (47.8 per cent) also received additional oral antibiotics. Corticosteroids (IV or oral) were administered in 42.9 per cent of patients (32.6 per cent in MG, 53.3 per cent in SG). The length of hospital stay was similar between MG and SG, with a median of 4 days (interquartile range [IQR]: 2.0-5.0).

For patient who underwent surgery, 92.7 per cent (*n* = 38) had a transoral approach, while 7.3 per cent (*n* = 3) underwent transcervical drainage. No patients required intubation prior to surgery, though six SG patients stayed intubated post-operatively for airway protection. The presence of pus intra-operatively was not associated with size or CSA of a collection.

Twenty-six patients (58.9 per cent) were initially treated using antibiotics and later required surgery due to clinical deterioration or insufficient improvement. The conversion to SG from MG happened at a median of two days after admission (IQR 1-3 days). On bivariate analysis, the single statistically significant symptom, fever, showcased an odds ratio of 11.72 (95% confidence interval [CI] 1.45–94.79). Additional bivariate analyses revealed that abscesses with a long-axis dimension measuring greater than 3.00 cm (odds ratio 8.89, 95% CI 1.56–50.53) and CSA greater than 3.00 cm^2^ (odds ratio 4.69, 95% CI 1.15–19.16) were relevant cutoffs to support the need of early surgical drainage (Table 5).

**Table 5.** Predictive factors for patient converting from medical to surgical treatment Table 5 long description.

Variable		Odds ratio	95% CI
Symptoms			
	Fever	11.72	1.45−94.79
Cross-section area			
	>2.00 cm^2^	6.00	0.65−55.62
	>2.50 cm^2^	5.19	0.95−28.5
	>3.00 cm^2^*	4.69	1.15−19.16
	>4.00 cm^2^	3.81	0.99−14.64
Largest long-axis dimension			
	>2.00 cm	2.25	0.55−9.18
	>2.50 cm	3.00	0.79−11.42
	>3.00 cm*	8.89	1.56−50.53
	>4.00 cm	4.50	0.42−47.76

CI = confidence interval.

* Denotes significance.

Complications in hospital (n = 7) were uncommon (Table 6). Two patients in the surgical group required repeat surgery for symptom relief. There were no cases of mortality across cohort.

**Table 6.** Identified complications during treatment and course of hospital admission Table 6 long description.

Treatment group	Complications	Outcome
Converted from medical to surgical	Post-extubation agitation, hematuria post-catheter removal, transient pancytopenia	Supportive care
Surgical	Incomplete drainage of parapharyngeal abscess	Repeat surgery
Surgical	Persistent symptoms following surgery	Repeat surgery
Surgical	Unable to void post-operatively	In/out catheter insertion
Surgical	Increased C-reactive protein following surgery, stridor	Intravenous dexamethasone and supportive care
Medical	Rash after clindamycin	Supportive care
Medical	Rash after Computed tomography	Supportive care

## Discussion

The management of paediatric DNSI remains challenging, largely resulting from consistent findings of variable, non-specific clinical presentation. Within this cohort, our study found that younger age, fever on presentation, CT-confirmed abscess and collection CSA greater than 3.00 cm^2^ were associated with an increased likelihood of surgical intervention. These findings support the role of conservative therapy in many cases while identifying factors that may warrant earlier surgical consideration.

Within our cohort, younger children were more likely to require surgery, reflecting the persistence of reactive lymphoid tissue in the retropharyngeal space during early childhood that can predispose them to more aggressive infections.^1^^,^^2^^,^^20^ The presence of fever on presentation emerged as another significant predictor, suggesting that fever serves as a nonspecific marker of systemic inflammatory response rather than a direct indicator of disease severity. In contrast, symptoms such as neck stiffness, sore throat or decreased oral intake which appear to be intuitive markers of severity, did not predict outcomes in our cohort. Additionally, our data showed that laboratory findings such as C-reactive protein levels, did not provide utility to determine management choice. These findings are consistent with prior studies that demonstrate a lack of reliable correlation between presenting clinical signs/symptoms and surgical necessity.^3^^,^^12^^,^^21^ The prognostic uncertainty underscores the difficulty of relying on clinical features alone.

CT findings remain central to diagnosis and are frequently cited as predictors of intervention.^3^^,^^4^^,^^6^^,^^9^^,^^14^^,^^21^ Within our series, abscess was more commonly classified within the surgical group. Beyond abscesses, those who required surgical drainage also had CT report of suppurative lymph node (15.9 per cent), oedema (9.1 per cent) and phlegmon (4.5 per cent). In comparison, 35.4 per cent of those with an initial diagnosis of abscess were also treated successfully by medical treatment alone. These findings are consistent with prior reports demonstrating substantial overlap in treatment outcome when considering CT features, highlighting that CT may overestimate the need for drainage and should be interpreted in conjunction with clinical finding.^14^

The size of a collection on imaging for DNSI has been a frequently utilised predictor for surgical intervention. Our study examined the largest long-axis dimension, which allows our findings to be compared directly to the existing literature. Previous studies have suggested thresholds ranging from greater than 1.5 cm to greater than 3.0 cm for surgical intervention:^3^^,^^4^^,^^12^^,^^22^ Wilkie *et al*. identified diameter of 2.5 cm as a highly predictive cutoff (area under the curve 0.85),^22^ while Wong *et al*. reported 25 mm as clinically relevant.^12^ Within our cohort, a cutoff of 3.00 cm in largest length measurement and 3.00 cm^2^ in CSA was suggestive as a threshold for consideration of surgery. While the CSA calculation cannot account for the irregular shape of abscesses and may under- or overestimate true size, it provides a practical benchmark for clinical decision-making compared to more complex methods, such as volumetric segmentation or geometric modelling. Using the readily reported length, width or height provides a practical estimate that can be calculated across patients and easily communicated between teams. Furthermore, some studies have found that RE has high utility for identifying purulent infections,^3^^,^^9^^,^^23^ whereas other studies describe the findings to be non-specific.^6^^,^^24^ Within our series, RE was commonly identified but not independently predictive of surgery after accounting for size.

Overall, more than half of the patients were managed successfully with antibiotics, which was higher than the previously reported range of 12-35 per cent.^3^^,^^4^^,^^25^ It should be noted that some previous studies focused on abscesses,^1^^,^^3^^,^^4^^,^^7^^,^^11^^,^^14^^,^^15^^,^^26^^,^^27^ at times due to constraints of available imaging modalities, our study also included infections with lower acuity such as cellulitis, suppurative lymph node and oedematous lymph nodes. For our cohort, the subset of patients who received initial medical therapy and later underwent surgical drainage provides important insight into treatment outcome. Twenty-six patients (35.6 per cent of all medically treated patients and 57.8 per cent of total surgically treated) required conversion to surgery, typically within 2-days after medical therapy. Fifteen patients within this group (57.7 per cent) were originally diagnosed with an abscess on CT. Among them, the size of the collection with long axis greater than 3.00 cm had a odds ratio of 8.89 (95% CI: 1.56-50.53) and CSA greater than 3.00 cm^2^ (odds ratio 4.69, 95% CI 1.15-19.16) for conversion to surgery. This suggests long-axis and CSA cutoffs can be used pre-emptively to identify patients who may eventually require surgery. Importantly, complication rates were not higher among converted cases compared to those undergoing early surgery. These findings suggest conservative management in stable patients is generally safe, provided close inpatient monitoring is available for prompt conversion where necessary.^3^^,^^4^

Corticosteroid use has been shown to be safe, lead to symptomatic improvement and decrease the length of stay.^27^ Within the study cohort, corticosteroid use was more commonly used within the surgical group (53.3 per cent). This likely reflects selection bias, where patients with more severe presentations received corticosteroids to reduce inflammation or avoid airway complications. Previous studies by Goenka *et al*. and Tansey *et al*. reported that corticosteroid administration was associated with lower odds of surgical drainage.^15^^,^^28^ However, their findings were also associated with higher rates of readmission for delayed surgeries on return to the hospital. The authors hypothesise that corticosteroids can alleviate acute symptoms, but such relief may only be temporary, while potentially creating a theoretical risk of masking the progression of the infection.^15^^,^^28^ Therefore, corticosteroids should be used as an adjunct to, rather than a substitute for, drainage when clinically indicated.

This study has several limitations. As a retrospective, single-centre review, it is subject to selection bias and variability in documentation. Details about patients’ fever before presentation, such as the duration of fever, as well as use of and response to antipyretics were not consistently recorded and could not be accurately obtained. The sample size limited subgroup analyses, and inclusion of both abscess and non-abscess entities (phlegmon, cellulitis suppurative lymphadenitis) introduces heterogeneity that may influence outcomes. Despite this heterogeneity, DNSI carries significant variation in presentation and infections of differing acuities can look the same. CSA was estimated using the two largest long-axis dimensions, a practical but less precise method than volumetric techniques. Management decisions such as timing of surgery, antibiotic choice and corticosteroid use were made at provider discretion, introducing further variability. Prospective multicentre trials are needed to further characterise features of fever, compare early surgery against antibiotic-first strategies and validate standardised CT reporting.

Despite these limitations, our study provides valuable insights. The cohort spans across a decade and represents one of the larger Canadian paediatric series on DNSI. Our study also provided valuable insight to suggest that initial medical treatment with antibiotics can be safe with appropriate monitoring. Even patients who required conversion to surgery did not experience complication despite some delay to drainage. Despite limitations of CSA measurements, it mirrors measurement approaches in literature and real-world practice for clinicians who rely on routinely reported dimensions for informed decision-making. Importantly, our findings align with existing literature, reinforcing abscess size, fever and younger age as reproducible predictors of surgical intervention. By adding these results to the existing literature, our study supports to the development of more standardised, evidence-based algorithms for paediatric retropharyngeal and parapharyngeal abscesses.

Ongoing research will be important for further characterisation of management decisions for DNSI. Additionally, given the heterogeneity of abscess and non-abscess entities in DNSI, prospective studies with larger, stratified cohorts will be valuable for accurate, individualised conclusions in management. Future research should also examine evolving microbiology, cost-effectiveness and quality of life outcomes of DNSI to characterise granular insights within this condition.

- Retropharyngeal and parapharyngeal infections in children can be life-threatening, with ongoing debate regarding indications for surgical drainage versus medical management alone
- Previous studies have suggested abscess size and rim enhancement on computed tomography (CT) may predict surgical need, but findings are inconsistent, and conversion rates from medical to surgical management remain under-reported
- Younger age, fever at presentation, CT-diagnosed abscess and larger cross-sectional area (>3.00 cm^2^) were independent predictors of surgical intervention while rim enhancement alone was not predictive of surgery when abscess size was considered
- A total of 51.1 per cent of CT-defined abscesses resolved with antibiotics alone, supporting conservative management in stable children
- These results provide evidence for use of imaging to guide clinical decision-making and highlight the need for larger multi-centre studies to validate predictors and establish standardised treatment guidelines

## Conclusion

Paediatric deep neck space infections have highly varied presentations and carry a risk of serious complications, necessitating timely and evidence-informed management. Within this study, younger age, fever on presentation, CT-confirmed abscess and cross-sectional area greater than 3.00 cm^2^ were common factors identified among patients that required surgical management. These findings contribute to a growing body of evidence that can inform clinical decision-making and guide treatment pathways. Future studies involving larger, multi-center cohorts are warranted to validate these prognostic markers and develop standardised management algorithms.

## Table 1 Long description

The table compares patient characteristics and medical history between medical and surgical groups. The surgical group has a significantly lower median age of 3.3 years compared to 5.1 years in the medical group, with a p-value of 0.0018. Sex distribution shows no significant difference, with males comprising 61.5% of the total. History of DNSI is absent in all patients. Recent URTI is more common in the surgical group (50%) than the medical group (29.6%), with a p-value of 0.0500. Prior antibiotic use and immunisation status show no significant differences between groups. Underlying health conditions are more prevalent in the medical group (23.9%) compared to the surgical group (11.4%), but this difference is not statistically significant.

Navigate back to Table 1.

## Table 2 Long description

The table compares symptoms and physical exam findings between medical and surgical groups. Fever is significantly more prevalent in the surgical group (91.1%) compared to the medical group (68.1%), with a p-value of 0.0092. Conversely, neck pain is more common in the medical group (63.8%) than in the surgical group (37.8%), with a p-value of 0.0125. Other symptoms like restriction of neck movement, decreased oral intake, and sore throat show no significant differences between the groups. Physical exam findings such as cervical lymphadenopathy and restricted neck mobility are similar across both groups. The data suggests specific symptoms like fever and neck pain may differentiate between the medical and surgical groups, but most other symptoms and findings do not show significant differences.

Navigate back to Table 2.

## Table 3 Long description

The table compares CT imaging features between medical and surgical cases, focusing on diagnosis categories. Abscesses are significantly more prevalent in surgical cases (68.9%) compared to medical cases (36.2%), with a p-value of 0.0048. Oedema is more common in medical cases (31.9%) than surgical cases (8.9%). Suppurative lymph nodes and phlegmon show minor differences between groups, while cellulitis is only present in medical cases (8.5%). The p-value indicates statistical significance in the distribution of diagnosis categories between medical and surgical groups.

Navigate back to Table 3.

## Table 4 Long description

The table compares CT imaging characteristics between medical and surgical groups, focusing on size, cross-sectional area (CSA), and rim enhancement. The surgical group has a larger median CSA of 5.0 cm² compared to 2.6 cm² in the medical group, with significant differences for dimensions over 2.50 cm² (p < 0.05). The largest long-axis dimension is slightly higher in the surgical group, but not significantly different. Rim enhancement is more common in the surgical group (90.6%) than in the medical group (72.7%). The data suggests a trend towards larger and more enhanced features in the surgical group, with some statistically significant differences.

Navigate back to Table 4.

## Table 5 Long description

The table evaluates predictive factors for patients transitioning from medical to surgical treatment, focusing on symptoms, cross-section area, and largest long-axis dimension. Fever has the highest odds ratio of 11.72, indicating a strong association with surgical conversion. Cross-section areas greater than 3.00 cm2 and largest long-axis dimensions greater than 3.00 cm are also significant, with odds ratios of 4.69 and 8.89, respectively. Other measurements show varying odds ratios, but only these three factors are statistically significant. The confidence intervals suggest variability in the data, and caution should be taken when interpreting non-significant results.

Navigate back to Table 5.

## Table 6 Long description

The table compares complications and outcomes across different treatment groups during hospital admission. Surgical treatments frequently resulted in repeat surgeries due to issues like incomplete drainage and persistent symptoms. In contrast, medical treatments primarily led to supportive care, particularly for rashes following clindamycin and CT scans. Notably, the conversion from medical to surgical treatment resulted in multiple complications, managed with supportive care. The data suggests surgical interventions may lead to more severe complications requiring further surgical intervention, while medical treatments tend to result in less severe complications managed with supportive care.

Navigate back to Table 6.
